# Physical Activity and the Development of Self-Regulation in Early Childhood: A Systematic Review and Meta-Analysis

**DOI:** 10.3390/jintelligence14070121

**Published:** 2026-06-25

**Authors:** Daniela Cecic-Mladinic, María del Carmen Carcelén-Fraile, Diana Marcela Aristizábal-García, Noelia Vigil-Torres

**Affiliations:** 1 Department of Educational Sciences, Faculty of Social Sciences, University of Atlántico Medio, 35017 Las Palmas de Gran Canaria, Spain; 2 International Scientific Association on Innovation in Education and Health (ACIINES), 23007 Jaen, Spain; 3 International Network of Educational Law, 35017 Las Palmas de Gran Canaria, Spain; 4 Faculty of Human and Social Sciences, University of San Buenaventura-Cali, Santiago de Cali 760016, Colombia

**Keywords:** physical activity, self-regulation, executive function, early childhood, meta-analysis, systematic review

## Abstract

Self-regulation is a foundational developmental skill in early childhood that supports academic readiness, social competence, and long-term health outcomes. Physical activity has been proposed as a modifiable behavior that may contribute to self-regulation development; however, evidence in preschool-aged children remains inconsistent and has primarily focused on cognitive outcomes. This systematic review and meta-analysis aimed to examine the association between physical activity and self-regulation across domains in early childhood using cross-sectional evidence. The review was conducted in accordance with PRISMA guidelines and registered in PROSPERO (CRD420261361512). Searches were conducted in PubMed, ERIC, CINAHL, and Web of Science. Fifteen studies met the inclusion criteria and were included in the qualitative synthesis and meta-analysis. A random-effects meta-analysis revealed a small but statistically significant positive association between physical activity and self-regulation (r = 0.099, 95% CI = 0.064–0.133, *p* < 0.001), although substantial heterogeneity was observed (I^2^ = 91.1%). Subgroup analyses showed significant associations for both cognitive and non-cognitive self-regulation outcomes. Sensitivity analyses confirmed the robustness of the findings, and no substantial publication bias was detected. Physical activity may represent a promising modifiable factor that supports self-regulation development in early childhood, although longitudinal and experimental research is needed to clarify causal relationships.

## 1. Introduction

Self-regulation, broadly defined as the ability to monitor and modulate cognition, emotions, and behavior in pursuit of goal-directed actions, is considered a foundational developmental skill established during early childhood (Day et al., 2022). Self-regulation encompasses multiple interrelated domains, including cognitive processes such as attention control, working memory, and inhibitory control, as well as emotional and behavioral regulation (Hosch et al., 2022; Janicaud et al., 2026). These processes enable children to manage impulses, sustain attention, adapt behavior to contextual demands, and engage in socially appropriate and goal-directed responses (Yusumut et al., 2026).

The development of self-regulation during the preschool years has been consistently linked to a broad range of positive developmental outcomes, including school readiness, academic achievement, social competence, and long-term physical and mental health (Nilfyr & Ewe, 2025). Children with stronger self-regulatory skills in early childhood tend to demonstrate better classroom engagement, more adaptive peer interactions, and improved learning-related behaviors, while deficits in self-regulation have been associated with behavioral difficulties, poorer educational outcomes, and increased risk for adverse health trajectories later in life (Hosokawa et al., 2024).

Early childhood represents a particularly sensitive period for the development of self-regulatory processes due to the rapid maturation of neural systems underlying executive control and behavioral regulation, particularly within prefrontal brain networks (Hodel, 2018). During this period, self-regulation develops through dynamic interactions between biological maturation, environmental influences, and daily experiences that provide opportunities to practice regulatory skills (Meredith & Silvers, 2024). As a result, identifying modifiable factors that may support self-regulation development during early childhood has become an important research priority (Montroy et al., 2016).

Among modifiable factors that may support self-regulation development, physical activity has emerged as a behavior of increasing interest (Belcher et al., 2021). In early childhood, physical activity occurs largely through active play, outdoor play, structured movement opportunities, and participation in games that may place natural demands on children’s attention, inhibitory control, behavioral flexibility, and emotional regulation (McGowan et al., 2024). For example, games such as “Simon Says,” tag, obstacle courses, and team-based activities require children to follow rules, wait for turns, inhibit impulsive responses, and adapt their behavior to changing situations. Outdoor play may also encourage children to negotiate social interactions, manage frustration, assess risks, and regulate emotions while exploring dynamic environments. Likewise, movement-based pretend play often requires children to maintain roles, remember sequences of actions, and coordinate behavior with peers. These movement-related experiences may therefore provide meaningful opportunities to engage and practice self-regulatory skills in developmentally appropriate and ecologically valid contexts (Savina, 2025). Several mechanisms have been proposed to explain the potential association between physical activity and self-regulation (Doré et al., 2020). From a neurodevelopmental perspective, physical activity may support the maturation and functioning of brain networks involved in executive control, particularly those centered in the prefrontal cortex. Regular engagement in physical activity has been associated with increased cerebral blood flow, enhanced synaptic plasticity, and greater expression of neurotrophic factors, such as brain-derived neurotrophic factor (BDNF), which are thought to facilitate neural development and cognitive functioning. These neurobiological processes may contribute to improvements in executive functions that constitute key components of self-regulation, including attentional control, inhibitory control, working memory, and cognitive flexibility.

Physical activity may also support self-regulation through behavioral and experiential pathways. Many movement-based activities require children to focus attention on task demands, inhibit automatic responses, remember rules, and adapt their behavior to changing situations. For example, games involving turn-taking, rule-switching, or coordinated group participation place direct demands on executive control processes. Repeated engagement in these activities may provide opportunities to practice and strengthen self-regulatory skills in naturalistic contexts. Consequently, physical activity may influence self-regulation through the combined effects of neurobiological adaptations and repeated behavioral practice of executive control processes during everyday movement experiences.

Despite growing evidence linking physical activity to self-regulation across older children, adolescents, and adults (Robinson et al., 2016; Yang et al., 2025; Umstattd et al., 2009), the nature of this association during early childhood remains less clearly understood. Existing evidence in preschool-aged children has produced mixed findings, and prior syntheses have been limited in scope. Earlier systematic reviews focusing on young children primarily examined associations between physical activity and cognitive dimensions of self-regulation, such as attention, working memory, inhibitory control, and cognitive flexibility, with some reporting positive associations and others suggesting inconsistent findings across specific executive function components (Wood et al., 2020; Carson et al., 2016). However, these reviews have largely focused on cognitive self-regulation and have not comprehensively addressed the broader multidimensional nature of self-regulation, which also includes emotional and behavioral domains. As a result, the extent to which physical activity may be associated with self-regulation across domains during early childhood remains insufficiently understood. In addition, previous evidence syntheses have not specifically provided a quantitative meta-analytic synthesis focused exclusively on cross-sectional evidence, nor have they adequately addressed the consistency of observed associations across studies.

Given these gaps, a comprehensive synthesis focusing on associations between physical activity and self-regulation across domains in early childhood is warranted. The present systematic review and meta-analysis therefore aimed to examine the association between physical activity and self-regulation in early childhood using evidence from cross-sectional studies. It was hypothesized that higher levels of physical activity would be positively associated with stronger self-regulation.

## 2. Materials and Methods

### 2.1. Study Design

This systematic review and meta-analysis were conducted according to the PRISMA guidelines (Page et al., 2021) (Supplementary Material) and prospectively registered in PROSPERO (registration number: CRD420261361512). The review aimed to examine the association between physical activity and self-regulation in early childhood using evidence from cross-sectional studies.

For the purposes of this review, physical activity was defined as any bodily movement produced by skeletal muscles that results in energy expenditure. Consistent with this broad definition, studies were eligible if they assessed any form of physical activity or movement behavior during early childhood, including total physical activity, light-, moderate-, or vigorous-intensity physical activity, moderate-to-vigorous physical activity, active play, outdoor play, structured movement activities, or sports participation. No minimum intensity threshold was required for inclusion, as the review aimed to capture the full range of physical activity exposures examined in relation to self-regulation outcomes in young children.

### 2.2. Eligibility Criteria

Studies were included if they: (1) involved children in early childhood (mean age ≤5 years); (2) measured physical activity, including duration or type (e.g., active play, outdoor play, various-intensity physical activity, total physical activity, or sports participation); (3) measured at least one domain of self-regulation (cognitive, emotional, or behavioral), or a global measure of self-regulation; (4) measured at least one of physical activity or self-regulation during early childhood, even if the second variable was measured at a later age; (5) used a cross-sectional design; (6) reported sufficient statistical data to calculate an effect size (e.g., correlation coefficients, standardized regression coefficients, odds ratios, or data convertible into effect sizes); and (7) were published in peer-reviewed journals, with no language restrictions applied.

Studies were excluded if they focused exclusively on physical fitness, involved exclusively children with diagnosed disabilities, developmental disorders, or clinical conditions, consisted of gray literature (e.g., dissertations, theses, conference proceedings, conference abstracts, reports, or unpublished materials), did not report relevant associations, or lacked sufficient data for meta-analysis.

### 2.3. Search Strategy

A systematic literature search was conducted in PubMed, ERIC, CINAHL, and Web of Science to identify studies published from 2020 to 2025. The search was designed to identify cross-sectional studies examining associations between physical activity and self-regulation in early childhood. The search terms combined MeSH terms and free-text terms related to physical activity, self-regulation, early childhood, and study design, using Boolean operators (AND, OR). The main search strategy was as follows: (“physical activity” OR “active play” OR “outdoor play” OR “sports participation” OR “moderate-to-vigorous physical activity” OR MVPA OR “total physical activity”) AND (“self-regulation” OR “self-regulation” OR “executive function” OR “inhibitory control” OR “behavioral regulation” OR “emotional regulation”) AND (“early childhood” OR preschool* OR preschooler* OR kindergarten OR “young child*”) AND (“cross-sectional” OR observational).

### 2.4. Study Selection Process

All references retrieved from the database searches were exported to EndNote, where duplicate entries were detected and removed prior to the screening process. The selection of studies followed a two-step procedure. Initially, titles and abstracts were reviewed according to the predefined inclusion and exclusion criteria, allowing the elimination of records that were clearly not relevant. Subsequently, the full texts of studies considered potentially eligible were obtained and examined in depth to determine their suitability for inclusion. Both the title/abstract screening and the full-text review were carried out independently by two researchers. Any discrepancies in eligibility decisions were discussed until agreement was reached, and a third reviewer was consulted when consensus could not be achieved. Exclusion decisions made during the full-text review were systematically documented and classified according to the underlying reason, such as unsuitable study design, inappropriate exposure or outcome variables, or inadequate information for calculating effect sizes. The study selection procedure was reported in accordance with PRISMA guidelines through a flow diagram. Ultimately, 15 studies satisfied all eligibility criteria and were included in both the qualitative synthesis and the meta-analytic evaluation.

### 2.5. Data Extraction

Two reviewers independently extracted the study data using a predefined and standardized extraction template. Information collected from each study comprised study characteristics (e.g., author, publication year, country, and study design), participant details (sample size, average age, and sex distribution), characteristics of the physical activity exposure (type, duration, intensity, and assessment method), self-regulation outcomes (specific domain evaluated and measurement tool used), statistical approaches, covariates considered in adjusted models, and the principal study results. When relevant information was incomplete, unavailable, or ambiguous, the corresponding authors were contacted to obtain clarification or additional data. Any inconsistencies between reviewers during the extraction process were discussed and resolved by agreement.

### 2.6. Risk of Bias Assessment

Methodological quality and risk of bias were evaluated independently by two reviewers using the Joanna Briggs Institute (JBI) Critical Appraisal Checklist for Analytical Cross-Sectional Studies (Moola et al., 2020). The assessment considered several methodological aspects, including the adequacy of inclusion criteria, the validity and reliability of exposure and outcome measurements, the identification and control of potential confounding variables, and the suitability of the statistical analyses performed. Each included study was examined across the eight domains of the JBI checklist and categorized according to the total number of criteria satisfied. Studies fulfilling seven or eight criteria were classified as having a low risk of bias, those meeting four to six criteria were considered to have a moderate risk of bias, and studies satisfying three or fewer criteria were classified as having a high risk of bias. Differences in reviewers’ judgments were addressed through discussion until consensus was achieved, with involvement of a third reviewer when required.

### 2.7. Analytical Decisions for Meta-Analysis

Several a priori analytical decisions were made to ensure consistency across studies and independence of effect sizes in the meta-analysis. Correlation coefficients were prioritized as the primary effect size metric, as they were considered the most appropriate measure for synthesizing associations reported in cross-sectional studies. When studies reported statistics in alternative formats, effect sizes were converted into correlation coefficients where possible and transformed into Fisher’s z values prior to analysis. When multiple effect sizes were reported within a study, a single effect size was selected for the primary analysis according to predefined decision rules to avoid violating assumptions of statistical independence. These decision rules were established a priori and applied consistently across all included studies. When both adjusted and unadjusted estimates were available, adjusted estimates were prioritized. When multiple physical activity exposures were reported, total physical activity was prioritized over specific subtypes unless examined separately in subgroup analyses. When multiple self-regulation outcomes were reported, global measures were prioritized; if unavailable, the outcome most closely reflecting cognitive self-regulation was selected. When studies reported multiple subgroup-specific associations (e.g., by sex), these were combined where appropriate to generate a single independent effect size per study. Additional effect sizes from the same study, when relevant, were explored in subgroup or sensitivity analyses. Meta-analyses were conducted using Comprehensive Meta-Analysis (Version4; Biostat, Englewood, NJ, USA) using a random-effects model to account for expected between-study heterogeneity. Pooled effect sizes were transformed back into correlation coefficients for interpretation. Specifically, the hierarchy followed was: (1) adjusted estimates were prioritized over unadjusted estimates; (2) total physical activity was prioritized over specific physical activity subtypes; (3) global self-regulation measures were prioritized over domain-specific outcomes; and (4) when no global measure was available, the outcome most closely reflecting cognitive self-regulation was selected.

Effect sizes were synthesized using a random-effects meta-analytic framework, which was selected to accommodate anticipated methodological and statistical variability among the included studies. Prior to aggregation, correlation coefficients were converted to Fisher’s z scores to improve statistical properties during analysis; pooled estimates were subsequently reconverted into correlation coefficients to facilitate interpretation. Between-study heterogeneity was evaluated using both Cochran’s Q test and the I^2^ index. Consistent with conventional thresholds, I^2^ values of approximately 25%, 50%, and 75% were interpreted as indicating low, moderate, and substantial heterogeneity, respectively. To examine the robustness of the findings, sensitivity analyses were performed using a leave-one-study-out procedure, whereby each study was sequentially removed and the pooled estimate recalculated. Subgroup analyses were specified a priori and were informed by both theoretical and empirical considerations. Specifically, they were based on the multidimensional conceptualization of self-regulation, which distinguishes cognitive and non-cognitive domains, and on previous evidence suggesting that associations between physical activity and self-regulation may vary according to the specific outcomes assessed. Furthermore, subgroup analyses were intended to explore potential sources of heterogeneity arising from differences in outcome measurement and study characteristics.

When the number of available studies allowed, subgroup analyses were undertaken based on characteristics of the physical activity exposure, the specific self-regulation domain assessed, and the measurement approach employed. Potential publication bias was examined through visual evaluation of funnel plot symmetry and, where at least ten studies were included, by applying Egger’s regression test. Statistical significance was determined using a two-sided threshold of *p* < 0.05.

## 3. Results

### 3.1. Study Selection

**Figure 1:**
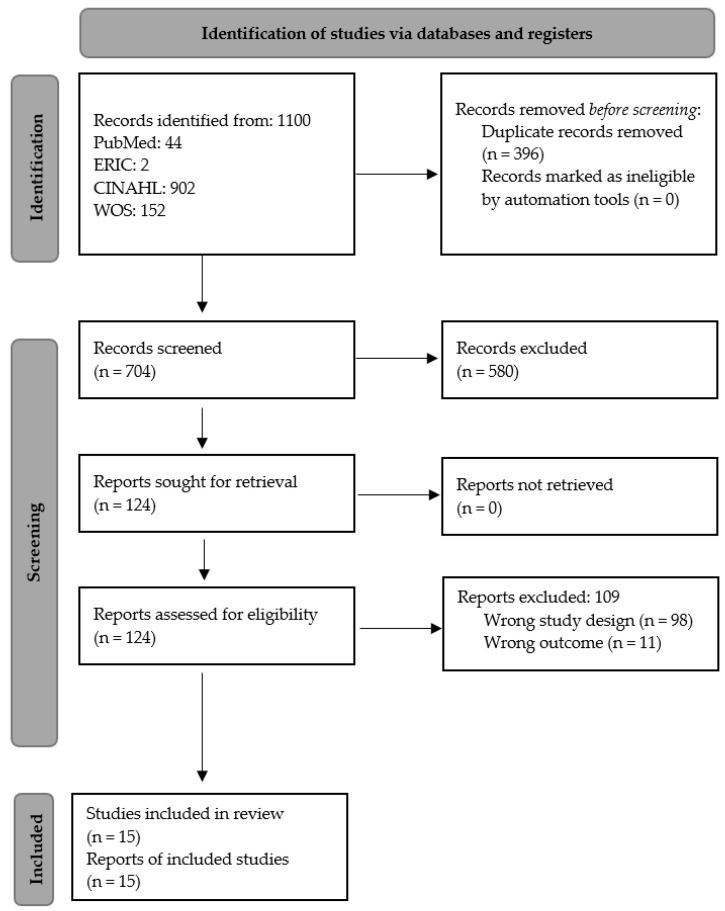
PRISMA 2020 flow diagram of the study selection process.

The database search yielded 1100 records from four electronic sources: PubMed (*n* = 44), ERIC (*n* = 2), CINAHL (*n* = 902), and Web of Science (*n* = 152). After removing 396 duplicate entries, 704 unique records remained for title and abstract screening. At this stage, 580 records were excluded because they did not meet the predefined eligibility criteria. A total of 124 full-text articles were subsequently retrieved and assessed for eligibility. Following detailed review, 109 articles were excluded, primarily due to an inappropriate study design (*n* = 98) or unsuitable outcome measures (*n* = 11). Consequently, 15 studies fulfilled all inclusion criteria and were retained for the systematic review and meta-analysis. The complete study selection procedure is illustrated in the PRISMA flow diagram presented in Figure 1.

### 3.2. Study Characteristics

The characteristics of the 15 included studies are summarized in Table 1. Studies were published between 2017 and 2025, with the majority published from 2020 onwards, reflecting growing research interest in associations between physical activity and self-regulation during early childhood (T. A. Bezerra et al., 2021; T. Bezerra et al., 2022; Bryant et al., 2021; Carson et al., 2017; Cook et al., 2019, 2021; Dealey & Stone, 2018; García-Alonso et al., 2025; Kuzik et al., 2020; Leppänen et al., 2021; Luo et al., 2023; McGowan et al., 2023; Vabø et al., 2022; Vanhala et al., 2022; Willoughby et al., 2018).

Included studies were conducted across diverse geographical settings. In Europe, studies were conducted in Spain (García-Alonso et al., 2025), Finland (Leppänen et al., 2021; Vanhala et al., 2022), and Norway (Vabø et al., 2022). In Australia, studies included the work of Dealey and Stone (2018). In Asia, studies were conducted in China (Luo et al., 2023). In North America, studies included Carson et al. (2017), Kuzik et al. (2020), Bryant et al. (2021), McGowan et al. (2023), and Willoughby et al. (2018). In Africa, studies were conducted in South Africa (Cook et al., 2019, 2021; Draper et al., 2020). In South America, studies included T. A. Bezerra et al. (2021) and T. Bezerra et al. (2022), both conducted in Brazil. Sample sizes varied considerably across studies, ranging from relatively small preschool cohorts to larger population-based samples.

All included studies used observational cross-sectional designs, consistent with the review eligibility criteria. Participants were preschool-aged children, with ages generally ranging from 3 to 5 years, although some studies included slightly broader early childhood ranges (e.g., 0–5 years or up to 6 years). Of the 15 included studies, the majority (*n* = 12) focused specifically on children aged 3–5 years, while three studies (*n* = 2) included broader preschool-age ranges extending to 6 years and two studies (*n* = 1) included children within a wider early childhood range beginning before age 3.

Sample sizes varied considerably across studies, ranging from smaller cohorts of fewer than 100 participants to larger samples exceeding 1000 children. Ten studies (*n* = 10) included samples of fewer than 300 participants, two studies (*n* = 2) included between 300 and 600 participants, and three studies (*n* = 3) included samples larger than 600 participants. This variability reflects differences in study design, recruitment strategies, and whether studies were based on community cohorts, preschool-based samples, or larger population-based datasets.

### 3.3. Risk of Bias

Physical activity exposures varied across studies and included total physical activity, moderate-to-vigorous physical activity, active play, outdoor play, sports participation, motor competence-related movement behaviors, and 24-h movement behaviors incorporating physical activity, sedentary behavior, and sleep (T. A. Bezerra et al., 2021; Bryant et al., 2021; Kuzik et al., 2020; McGowan et al., 2023). Physical activity was assessed using both objective methods, such as accelerometry (e.g., T. A. Bezerra et al., 2021; Kuzik et al., 2020; Luo et al., 2023), and subjective methods, including parent-report and educator-report measures (e.g., Dealey & Stone, 2018).

Self-regulation outcomes were assessed across multiple domains, with the majority of studies examining cognitive self-regulation, particularly executive function components such as inhibitory control, working memory, attention, and cognitive flexibility (Cook et al., 2019; Luo et al., 2023; Vabø et al., 2022; Vanhala et al., 2022). Fewer studies examined broader behavioral or emotional regulation outcomes, or global self-regulation measures (Cook et al., 2021; Leppänen et al., 2021). A variety of assessment tools were used, including direct performance-based tasks, standardized tests, and proxy-reported measures.

Risk of bias was assessed across the 15 included studies using the Joanna Briggs Institute for analytical cross-sectional studies (Moola et al., 2020). Based on the predefined classification criteria, 14 studies were rated as having low risk of bias, including T. A. Bezerra et al. (2021), T. Bezerra et al. (2022), Bryant et al. (2021), Carson et al. (2017), Cook et al. (2019, 2021), García-Alonso et al. (2025), Kuzik et al. (2020), Leppänen et al. (2021), Luo et al. (2023), McGowan et al. (2023), Vabø et al. (2022), Vanhala et al. (2022), and Willoughby et al. (2018). One study was classified as having moderate risk of bias, including Dealey and Stone (2018).

Overall, most studies met criteria related to clearly defined inclusion criteria, appropriate measurement of exposures and outcomes, and suitable statistical analyses (e.g., Carson et al., 2017; Kuzik et al., 2020; Luo et al., 2023; Vanhala et al., 2022). The most common methodological limitations were related to the identification and management of confounding factors, particularly the reporting of strategies used to address confounding (e.g., Dealey & Stone, 2018). These limitations were primarily observed in studies classified as having moderate risk of bias. Overall, the included evidence was considered to be of predominantly low methodological risk of bias. Detailed ratings for individual studies are presented in Table 2.

### 3.4. Results of the Meta-Analysis

**Figure 2:**
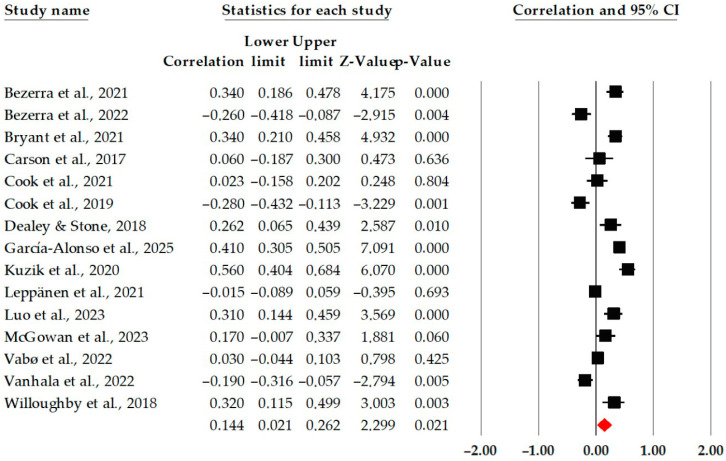
Forest plot of the association between physical activity and self-regulation/executive function in preschool children. Black squares represent individual study effect sizes, with square size proportional to study weight. Horizontal lines indicate 95% confidence intervals. The red diamond represents the pooled effect size and its 95% confidence interval. References for the studies included in the figure are provided in the reference list.

A total of 15 cross-sectional studies were included in the meta-analysis to examine the association between physical activity and self-regulation/executive function in preschool children. The heterogeneity analysis revealed a Q value of 157.634 with 14 degrees of freedom, indicating substantial between-study heterogeneity. Consistent with this, the I^2^ statistic was 91.1%, suggesting that a large proportion of the observed variability in effect sizes was due to true differences between studies rather than sampling error. Furthermore, the Tau-square and Tau values were 0.052 and 0.229, respectively, confirming considerable dispersion among the estimated true effects. Despite this heterogeneity, the pooled analysis showed a small but statistically significant positive association between physical activity and self-regulation/executive function (r = 0.099; 95% CI = 0.064–0.133; *p* < 0.001). Figure 2 presents the corresponding forest plot and illustrates the variability in effect sizes across the included studies.

#### 3.4.1. Subgroup Analyses by Self-Regulation Domain

Subgroup analyses were conducted to examine whether the association between physical activity and self-regulation differed according to the type of self-regulation outcome assessed. Studies were categorized into cognitive self-regulation outcomes (e.g., executive function, working memory, inhibitory control, and cognitive flexibility) and non-cognitive self-regulation outcomes (e.g., emotional regulation, behavioral regulation, and global self-regulation).

**Figure 3:**
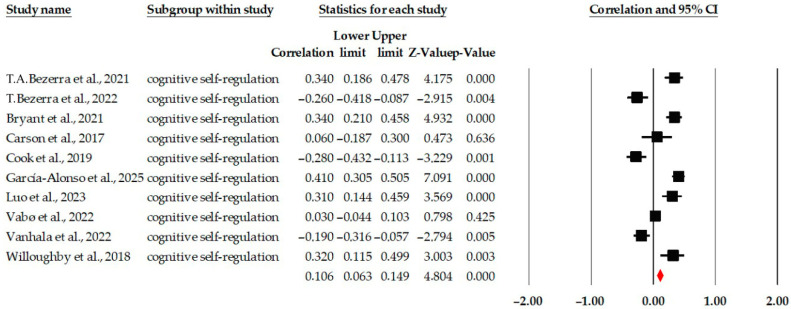
Forest plot of the subgroup meta-analysis examining the association between physical activity and cognitive self-regulation outcomes in preschool children. Black squares represent individual study effect sizes, with square size proportional to study weight. Horizontal lines indicate 95% confidence intervals. The red diamond represents the pooled effect size and its 95% confidence interval. References for the studies included in the figure are provided in the reference list.

For studies assessing cognitive self-regulation outcomes (k = 10), the pooled analysis revealed a small but statistically significant positive association between physical activity and cognitive self-regulation (r = 0.106; 95% CI = 0.063–0.149; *p* < 0.001). The corresponding forest plot is presented in Figure 3.

**Figure 4:**
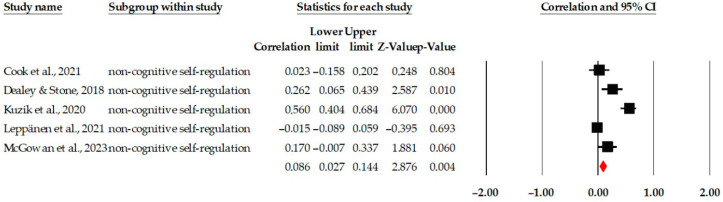
Forest plot of the subgroup meta-analysis examining the association between physical activity and non-cognitive self-regulation outcomes in preschool children. Black squares represent individual study effect sizes, with square size proportional to study weight. Horizontal lines indicate 95% confidence intervals. The red diamond represents the pooled effect size and its 95% confidence interval. References for the studies included in the figure are provided in the reference list.

Similarly, studies assessing non-cognitive self-regulation outcomes (k = 5) also showed a small but statistically significant positive association (r = 0.086; 95% CI = 0.027–0.144; *p* = 0.004), although substantial heterogeneity remained in this subgroup (I^2^ = 89.7%). The corresponding forest plot is presented in Figure 4. Overall, these findings suggest that physical activity may be positively associated with both cognitive and non-cognitive dimensions of self-regulation in preschool children, although the magnitude of these associations appears to be small.

#### 3.4.2. Publication Bias

**Figure 5:**
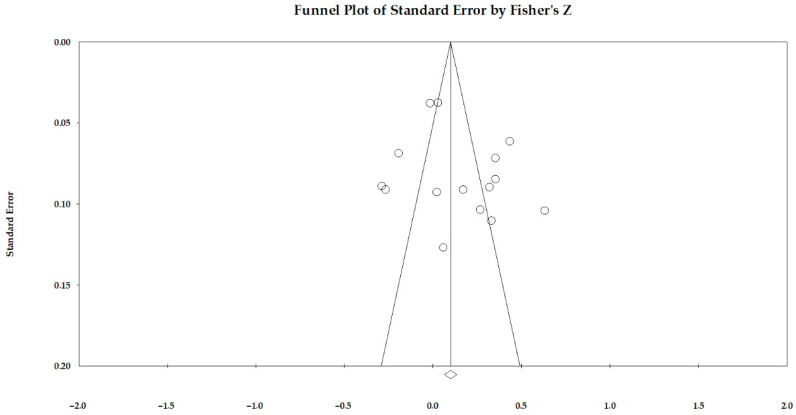
Funnel plot assessing publication bias across studies included in the meta-analysis. Open circles represent individual studies. The vertical line indicates the pooled effect estimate, and the diagonal lines represent the expected 95% confidence limits around the pooled effect.

Assessment of publication bias provided little indication of systematic distortion in the available evidence. Examination of the funnel plot revealed an overall pattern of study distribution that was largely symmetrical, despite the presence of minor visual asymmetry (Figure 5). Consistent with this observation, Begg and Mazumdar’s rank correlation test did not detect significant asymmetry (Kendall’s τ = 0.057, *p* = 0.766), and Egger’s regression test likewise produced a non-significant intercept (2.53, *p* = 0.280), suggesting the absence of meaningful small-study effects. The robustness of the pooled findings was further supported by the classic fail-safe N procedure, which indicated that an additional 155 unpublished studies with null results would be necessary to reduce the overall effect to non-significance. Furthermore, Duval and Tweedie’s trim-and-fill method estimated the possible absence of three studies; however, adjustment for these potentially missing studies did not alter the direction of the pooled effect, which remained positive. Taken together, these analyses suggest that the overall conclusions are relatively stable and unlikely to be substantially influenced by publication bias.

#### 3.4.3. Sensitivity Analysis

**Figure 6:**
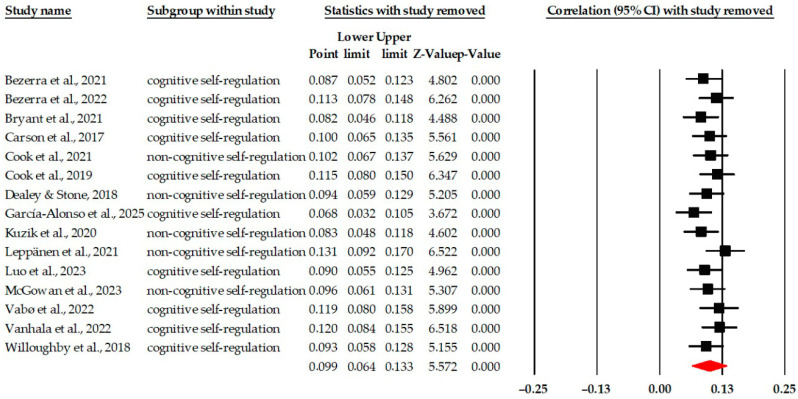
Leave-one-out sensitivity analysis of the association between physical activity and self-regulation. Black squares represent individual study effect sizes, with square size proportional to study weight. Horizontal lines indicate 95% confidence intervals. The red diamond represents the pooled effect size and its 95% confidence interval. References for the studies included in the figure are provided in the reference list.

A leave-one-out sensitivity analysis was conducted to assess whether the overall effect was disproportionately influenced by any individual study (Figure 6). The results showed that the pooled effect size remained stable after sequentially removing each study. Effect sizes ranged from r = 0.068 (after removing García-Alonso et al., 2025) to r = 0.131 (after removing Leppänen et al., 2021), and all pooled estimates remained statistically significant (all *p* < .001). These findings indicate that no single study exerted an undue influence on the overall meta-analytic effect, supporting the robustness of the results.

## 4. Discussion

The present systematic review and meta-analysis aimed to examine the association between physical activity and self-regulation in early childhood using evidence derived exclusively from cross-sectional studies. To our knowledge, this is the first meta-analysis to quantitatively synthesize the relationship between physical activity and multidimensional self-regulation outcomes, including cognitive, behavioral, emotional, and global self-regulation, specifically in preschool-aged children. Overall, the findings demonstrated a small but statistically significant positive association between physical activity and self-regulation (r = 0.099), suggesting that children who engage in higher levels of physical activity tend to exhibit slightly stronger self-regulatory capacities. Although the magnitude of this association was small, it remained statistically significant across analyses and was observed across both cognitive and non-cognitive self-regulation domains, reinforcing the potential developmental relevance of physical activity during early childhood.

The small magnitude of the pooled association should be interpreted cautiously but not dismissed as clinically irrelevant. Self-regulation is a complex developmental construct influenced by numerous biological, familial, educational, and environmental factors (Montroy et al., 2016; Meredith & Silvers, 2024). Consequently, it would be unrealistic to expect physical activity alone to explain large proportions of variance in self-regulatory functioning. Instead, the observed effect likely reflects the contribution of physical activity as one modifiable behavioral factor operating within a broader developmental system. Even small effects may be meaningful at a population level when considering that self-regulation in early childhood predicts later academic achievement, mental health, social competence, and physical health outcomes (Moffitt et al., 2011; Day et al., 2022; Hosokawa et al., 2024). From a public health and educational perspective, modest associations may still justify intervention efforts because physical activity is relatively low-cost, scalable, and associated with multiple additional health benefits.

Several mechanisms may explain the observed association between physical activity and self-regulation. First, neurobiological models suggest that movement behaviors may support maturation of neural systems involved in executive functioning, particularly within the prefrontal cortex. Physical activity has been associated with increased cerebral blood flow, improved neural connectivity, enhanced synaptic plasticity, and increased production of neurotrophic factors such as brain-derived neurotrophic factor (BDNF), all of which may contribute to executive control processes (Best, 2010; Diamond & Ling, 2016; Guzmán-Muñoz et al., 2025). Because the preschool years represent a period of rapid prefrontal development (Hodel, 2018), physical activity may be particularly influential during this developmental window. Second, behavioral and ecological explanations may also help explain the findings. Many forms of physical activity in early childhood involve structured games, free play, outdoor play, and sports participation, all of which require children to follow rules, wait for turns, inhibit impulsive responses, negotiate peer interactions, and adapt behavior to changing environmental demands (Lourenco & Casey, 2013; Nikolić et al., 2026). These contexts may function as natural opportunities for practicing self-regulation skills. For example, structured sports participation was positively associated with executive functioning in Bryant et al. (2021), while outdoor play opportunities were linked to behavioral regulation in Dealey and Stone (2018). These findings align with sociocultural theories suggesting that self-regulation develops through repeated participation in socially structured activities.

The subgroup analyses provide important insight into the multidimensional nature of self-regulation. Previous reviews have primarily focused on executive function outcomes such as inhibitory control, working memory, and attention (Carson et al., 2016; Wood et al., 2020). In contrast, the present review found significant associations not only for cognitive self-regulation outcomes but also for behavioral and emotional regulation outcomes. This is an important contribution because self-regulation in early childhood extends beyond executive functioning alone (Hosch et al., 2022). Physical activity may therefore support broader developmental competencies, including emotional adaptation, behavioral flexibility, and social functioning. However, the smaller number of studies examining non-cognitive domains highlights an important gap in the literature that should be addressed in future research.

Despite these promising findings, substantial heterogeneity was observed across studies (I^2^ = 91.1%), indicating considerable variability in effect sizes. Several factors likely contributed to this heterogeneity. First, studies used highly diverse physical activity measures, including accelerometer-based total physical activity, moderate-to-vigorous physical activity, active play, sports participation, and parent-reported movement behaviors. These different operationalizations may capture distinct dimensions of physical activity that are not equally related to self-regulation. For example, structured physical activity may place greater demands on executive control than unstructured movement. Second, substantial heterogeneity emerged from differences in self-regulation measurement. Included studies used direct cognitive tasks, parent reports, teacher reports, observational measures, and broader temperament assessments. These tools often assess overlapping but non-identical constructs and may differ in sensitivity, reliability, and ecological validity (Duckworth & Kern, 2011; Hosch et al., 2022). The conceptual fragmentation of self-regulation remains a major challenge in developmental research and likely contributed to between-study variability. Although these factors may represent important sources of heterogeneity, additional subgroup or moderator analyses based on specific physical activity exposures, measurement methods, or other study characteristics were not undertaken because many categories were represented by only a small number of studies. Consequently, such analyses would likely have produced unstable estimates and limited statistical power. Future reviews including a larger number of studies may be better positioned to formally examine these potential moderators and determine their contribution to between-study variability. Third, contextual differences may have influenced findings. Studies were conducted across diverse countries, including high-income and low- and middle-income settings. Cultural norms related to play, preschool curricula, outdoor access, parenting practices, and movement opportunities may shape both physical activity participation and self-regulatory development (Cook et al., 2019; Leppänen et al., 2021). These contextual factors remain underexplored and warrant greater attention. For example, access to safe outdoor play environments and green spaces may provide greater opportunities for active play, which in turn may support the development of attention regulation, behavioral control, and social competence (McCormick, 2017). Likewise, early childhood education settings differ substantially in the extent to which they incorporate outdoor learning, movement-based activities, and child-directed play, all of which may offer opportunities to practice self-regulatory skills. Cultural expectations regarding children’s autonomy, emotional expression, and behavioral control may also influence how self-regulation develops and how physical activity opportunities are structured within families and educational settings. Consequently, these contextual differences may partly explain the substantial variability observed across studies conducted in different countries and sociocultural environments.

An important issue that should be emphasized is the inability of cross-sectional studies to establish causal directionality. Although physical activity may enhance self-regulation, the reverse relationship is also plausible. Children with stronger self-regulatory capacities may be more likely to participate in organized activities, comply with structured movement opportunities, or persist in physically demanding tasks. Bidirectional associations are highly likely, and longitudinal studies are needed to clarify developmental pathways. Some intervention studies in related literature suggest that physically active interventions may improve executive functioning (Robinson et al., 2016), but stronger causal evidence in preschool populations remains limited.

Some longitudinal evidence supports this possibility. For example, Colliver et al. (2022) reported that greater participation in unstructured active play during the preschool years predicted stronger self-regulation two years later. Similarly, Howard et al. (2018) found evidence of bidirectional associations between sport participation and self-regulation in young children, showing that participation in sports predicted later self-regulation, while children with poorer self-regulation were less likely to engage in subsequent sport participation. Together, these findings suggest that physical activity and self-regulation may influence one another over time through reciprocal developmental processes. The findings should also be interpreted in relation to the broader 24-h movement behavior framework. Several included studies examined physical activity alongside sleep and sedentary behavior (T. A. Bezerra et al., 2021; Kuzik et al., 2020; McGowan et al., 2023), reflecting growing recognition that movement behaviors are interdependent. The 24-h movement behavior framework provides a useful perspective for understanding how physical activity may be situated within broader patterns of daily behavior. Although the present meta-analysis focused specifically on physical activity, previous research has suggested that physical activity, sleep, and sedentary behavior may interact in ways that influence child development. Consequently, future studies should examine the combined and potentially synergistic contributions of these movement behaviors to self-regulation development using longitudinal and compositional approaches. Future studies should increasingly adopt compositional and longitudinal approaches to better understand these interactions.

This review has several important strengths. It followed PRISMA guidelines, was prospectively registered in PROSPERO, included a rigorous risk of bias assessment, and incorporated both qualitative and quantitative synthesis. The inclusion of multiple self-regulation domains represents an important advancement beyond previous reviews focused solely on executive functioning. Additionally, publication bias analyses and sensitivity analyses supported the robustness of the findings. However, several limitations should be acknowledged. First, all included studies were cross-sectional, precluding causal inference. Second, substantial methodological heterogeneity limits the precision of pooled estimates. Third, the small number of studies examining emotional and behavioral self-regulation restricted subgroup comparisons. Fourth, some studies relied on parent-reported physical activity measures, which may introduce reporting bias. Finally, the unpublished literature and gray literature were not included, which may have increased the risk of publication bias despite statistical tests suggesting relative stability.

Future research should prioritize longitudinal and experimental designs to clarify causality and developmental mechanisms. Greater standardization of physical activity and self-regulation measurement is needed to improve comparability across studies. Researchers should also examine whether specific types of physical activity (e.g., structured sports, outdoor play, motor skill interventions, classroom movement programs) produce stronger self-regulatory benefits than total activity volume alone. Additionally, future work should explore moderators such as sex, socioeconomic status, neurodevelopmental profiles, childcare environments, and cultural contexts.

From a practical perspective, these findings support efforts to promote physically active opportunities during early childhood education and care settings. Preschool educators, families, and policymakers should recognize that movement may contribute not only to physical health but also to cognitive, behavioral, and emotional development. Integrating active play, outdoor learning, and structured movement opportunities into early childhood environments may represent a promising strategy to support holistic child development. Overall, this review provides emerging evidence that physical activity is positively associated with self-regulation in early childhood. Although the observed effects were modest and causal conclusions cannot yet be drawn, the findings reinforce the potential role of movement behaviors as an accessible and modifiable factor in early developmental health.

## 5. Conclusions

The findings of this systematic review and meta-analysis suggest that greater engagement in physical activity is associated with more favorable self-regulation outcomes during early childhood. Based on evidence from 15 cross-sectional studies, a small but statistically significant positive relationship was identified between physical activity and self-regulation, with consistent patterns observed across both cognitive and non-cognitive dimensions. These results indicate that movement-related behaviors may contribute to the development of executive functioning as well as behavioral and emotional regulation throughout the preschool period. Although the magnitude of the association was small, its potential relevance should not be overlooked given the well-established role of self-regulation in later educational achievement, health, and psychosocial adjustment. Nevertheless, several limitations warrant consideration. The substantial heterogeneity observed across studies, together with the exclusive reliance on cross-sectional evidence, restricts the ability to infer causality. Future investigations should emphasize longitudinal and experimental research designs to clarify the direction of these relationships and determine which characteristics of physical activity, including type, intensity, and context, are most strongly linked to self-regulation development. Improved consistency in assessment methods would also facilitate comparisons across studies and strengthen the evidence base. In summary, encouraging physical activity within early childhood education settings, family environments, and community contexts may represent a practical and potentially effective approach to supporting not only children’s physical well-being but also their cognitive, emotional, and behavioral development.

## Figures and Tables

**Table 1:** Characteristics of studies included in the systematic review.

Study	Country	Sample Size (*n*)	Design	Age Range	PA Measure	Self-Regulation Outcome
T. Bezerra et al. (2022)	Brazil	142	Cross-sectional	3–5 years	LPA + MPA + VPA-accelerometer	Executive function
T. A. Bezerra et al. (2021)	Brazil	123	Cross-sectional	3–5 years	MVPA-accelerometer	Executive function
Bryant et al. (2021)	USA	197	Cross-sectional	M_age_ = 4.34 years	Structured sports participation	Executive function
Carson et al. (2017)	Canada	100	Cross-sectional	30–59 months	TPA-accelerometer + parent report	Executive function
Cook et al. (2021)	South Africa	78	Cross-sectional	3–6 years	TPA-accelerometer	Executive function
Cook et al. (2019)	South Africa	268	Cross-sectional	3–6 years	TPA-accelerometer	Behavioral self-regulation
Dealey and Stone (2018)	Australia	93	Cross-sectional	3 years	TPA-survey	Behavioral self-regulation
García-Alonso et al. (2025)	Spain	268	Cross-sectional	4–6 years	Accelerometry	Executive function
Kuzik et al. (2020)	Canada	394	Cross-sectional	3–5 years	LPA + MVPA-accelerometer	Social–emotional regulation
Leppänen et al. (2021)	Finland	697	Cross-sectional	3–6 years	LPA + MPA + VPA + PA guidelines-accelerometer Physical activity + sedentary behavior	Temperament/self-regulation
Luo et al. (2023)	China	321	Cross-sectional	M_age_ = 4.4	Physical activity + fitness	Executive function
McGowan et al. (2023)	USA	123	Cross-sectional	M_age_ = 4.9 years	MVPA + PA—parent reported	Behavioral self-regulation
Vabø et al. (2022)	Norway	653	Cross-sectional	3–5 years	LPA + MPA + VPA + TPA-accelerometer	Executive function
Vanhala et al. (2022)	Finland	432	Cross-sectional	3–5 years	VPA-accelerometer	Executive function
Willoughby et al. (2018)	USA	407	Cross-sectional	3–5 years	Physical activity	Executive function

Note: TPA = total physical activity; LPA = light physical activity; MPA = moderate physical activity; VPA = vigorous physical activity; MVPA = moderate-to-vigorous physical activity; PA = physical activity. Studies were classified into cognitive self-regulation (e.g., executive function, inhibitory control, working memory, cognitive flexibility) and non-cognitive self-regulation (e.g., behavioral self-regulation, social–emotional regulation, temperament-related self-regulation) domains for subgroup analyses. Mean age is reported when age ranges were not available.

**Table 2:** Risk of bias assessment of included studies using the Joanna Briggs Institute Critical Appraisal Checklist for Analytical Cross-Sectional Studies.

Study	1	2	3	4	5	6	7	8	Total	Risk
T. Bezerra et al. (2022)	Yes	Yes	Yes	Yes	Yes	Yes	Yes	Yes	8/8	Low
T. A. Bezerra et al. (2021)	Yes	Yes	Yes	Yes	Yes	Yes	Yes	Yes	8/8	Low
Bryant et al. (2021)	Yes	Yes	Yes	Yes	Yes	No	Yes	Yes	7/8	Low
Carson et al. (2017)	Yes	Yes	Yes	Yes	Yes	Yes	Yes	Yes	8/8	Low
Cook et al. (2021)	Yes	Yes	Yes	Yes	Yes	No	Yes	Yes	7/8	Low
Cook et al. (2019)	Yes	Yes	Yes	Yes	Yes	Yes	Yes	Yes	8/8	Low
Dealey and Stone (2018)	Yes	Yes	Yes	Yes	Yes	No	Yes	No	6/8	Moderate
García-Alonso et al. (2025)	Yes	Yes	Yes	Yes	Yes	Yes	Yes	Yes	8/8	Low
Kuzik et al. (2020)	Yes	Yes	Yes	Yes	Yes	Yes	Yes	Yes	8/8	Low
Leppänen et al. (2021)	Yes	Yes	Yes	Yes	Yes	No	Yes	Yes	7/8	Low
Luo et al. (2023)	Yes	Yes	Yes	Yes	Yes	Yes	Yes	Yes	8/8	Low
McGowan et al. (2023)	Yes	Yes	Yes	Yes	Yes	No	Yes	Yes	7/8	Low
Vabø et al. (2022)	Yes	Yes	Yes	Yes	Yes	Yes	Yes	Yes	8/8	Low
Vanhala et al. (2022)	Yes	Yes	Yes	Yes	Yes	Yes	Yes	Yes	8/8	Low
Willoughby et al. (2018)	Yes	Yes	Yes	Yes	Yes	Yes	Yes	Yes	8/8	Low

Note. 1 = Inclusion criteria clearly defined; 2 = Study subjects and setting described; 3 = Exposure measured validly and reliably; 4 = Standard criteria used for outcome measurement; 5 = Confounding factors identified; 6 = Strategies to deal with confounding stated; 7 = Outcomes measured validly and reliably; 8 = Appropriate statistical analysis. Studies meeting 7–8 criteria were classified as low risk of bias, studies meeting 4–6 criteria as moderate risk of bias, and studies meeting 0–3 criteria as high risk of bias.

## Data Availability

Not applicable.

## Supplementary Materials

The following supporting information can be downloaded at: https://www.mdpi.com/article/10.3390/jintelligence14070121/s1, Supplementary Table S1. PRISMA 2020 Checklist.

## Abbreviations

The following abbreviations are used in this manuscript:

PA	Physical Activity
TPA	Total Physical Activity
LPA	Light Physical Activity
MPA	Moderate Physical Activity
VPA	Vigorous Physical Activity
MVPA	Moderate-to-Vigorous Physical Activity
SR	Self-Regulation
EF	Executive Function
PRSIMA	Preferred Reporting Items for Systematic Reviews and Meta-Analyses
PROSPERO	International Prospective Register of Systematic Reviews
JBI	Joanna Briggs Institute
CI	Confidence Interval
BDNF	Brain-Derived Neurotrophic Factor
OR	Odds Ratio
SD	Standard Deviation
